# 3D Kinect Camera Scheme with Time-Series Deep-Learning Algorithms for Classification and Prediction of Lung Tumor Motility

**DOI:** 10.3390/s22082918

**Published:** 2022-04-11

**Authors:** Utumporn Puangragsa, Jiraporn Setakornnukul, Pittaya Dankulchai, Pattarapong Phasukkit

**Affiliations:** 1 Division of Radiation Oncology, Department of Radiology, Faculty of Medicine, Siriraj Hospital, Mahidol University, Bangkok 10700, Thailand; utumporn.pua@mahidol.ac.th (U.P.); jiraporn.set@mahidol.ac.th (J.S.); pittaya.dan@mahidol.ac.th (P.D.); 2 School of Engineering, King Mongkut’s Institute of Technology Ladkrabang, Bangkok 10520, Thailand

**Keywords:** deep learning, Kinect V.2, 3D camera, times-series deep learning, lung cancer, external surrogate

## Abstract

This paper proposes a time-series deep-learning 3D Kinect camera scheme to classify the respiratory phases with a lung tumor and predict the lung tumor displacement. Specifically, the proposed scheme is driven by two time-series deep-learning algorithmic models: the respiratory-phase classification model and the regression-based prediction model. To assess the performance of the proposed scheme, the classification and prediction models were tested with four categories of datasets: patient-based datasets with regular and irregular breathing patterns; and pseudopatient-based datasets with regular and irregular breathing patterns. In this study, ‘pseudopatients’ refer to a dynamic thorax phantom with a lung tumor programmed with varying breathing patterns and breaths per minute. The total accuracy of the respiratory-phase classification model was 100%, 100%, 100%, and 92.44% for the four dataset categories, with a corresponding mean squared error (MSE), mean absolute error (MAE), and coefficient of determination (R^2^) of 1.2–1.6%, 0.65–0.8%, and 0.97–0.98, respectively. The results demonstrate that the time-series deep-learning classification and regression-based prediction models can classify the respiratory phases and predict the lung tumor displacement with high accuracy. Essentially, the novelty of this research lies in the use of a low-cost 3D Kinect camera with time-series deep-learning algorithms in the medical field to efficiently classify the respiratory phase and predict the lung tumor displacement.

## 1. Introduction

Every year, lung cancer claims an estimated 1.8 million lives worldwide [1]. The most common treatment options for lung cancer include surgery, chemotherapy, radiotherapy, and a combination of these treatments. Of particular interest is radiotherapy, which is normally adopted as an alternative treatment for surgically inoperable cancer or as a pre- or post-surgery routine [2].

Radiotherapy is a cancer treatment that uses high doses of radiation to kill cancer cells and shrink tumors. Specifically, radiotherapy involves delivering a high radiation dose to destroy the cancer while sparing the nearby healthy tissue [3,4]. In the course of radiation treatment of lung cancer, respiration-induced cancer motion could bring about distortion in the target cancer volume [5], a non-uniform radiation dose distribution, and, subsequently, ineffective radiation therapy [6] in addition to imaging artifacts (an image artifact is any feature that appears in an image that is not present in the original imaged object) [5,6,7,8,9]. As a result, respiration-induced motion management should be adopted for tumors with displacement (tumor motion) greater than 5 mm along the longitudinal axis (head–toe direction) or on all human anatomical axes [10]. 

Several respiration-induced motion management techniques have been proposed to characterize the tumor motion. Of particular interest is the four-dimensional computed tomography (4D CT) technique, which is commonly deployed to characterize the respiration-induced movement of internal organs and improve the quality of 3D CT images and tumor localization accuracy [11]. The generation of 4D CT images requires a CT simulator and a medical external surrogate device. A CT simulator is a CT scan machine capable of capturing images used in the planning of radiation therapy, and a medical external surrogate device is a system for tracking the breathing motion, thus enabling oncologists to visualize the tumor as it moves while a patient is breathing (i.e., respiratory phases) [12]. 

Currently, there exist several commercial medical external surrogate devices to track the respiration-induced chest wall movement [13], e.g., the real-time position management (RPM) system [14,15], the laser-based Sentinel™ surrogate device [16], and the AZ-733V flexible pressure belt to be placed on the patient’s chest wall [17]. However, the commercially available medical external surrogate devices are costly and of closed-system proprietary technology, thereby prohibiting modifications to the manufacturer settings and configurations.

In this study, we replaced the costly RPM system (i.e., a medical external surrogate device) with a highly efficient and budget-friendly off-the-shelf Kinect v2 3D camera. The Kinect v2 3D camera is an economical time-of-flight camera capable of detecting respiratory motion as an external surrogate in radiotherapy [18,19,20].

Furthermore, to streamline and improve the respiratory-phase classification and the prediction of lung tumor displacement, we incorporated two time-series deep-learning algorithmic models into the proposed Kinect camera scheme (i.e., the time-series deep-learning Kinect camera scheme). The proposed time-series deep-learning algorithmic models include a classification model to classify the respiratory phases with a lung tumor and a regression-based prediction model to predict the lung tumor displacement. More importantly, the time-series deep-learning classification and regression-based prediction models are both of an open-system algorithmic scheme as opposed to the closed-system technology of the commercial external surrogate devices, e.g., the RPM system.

Specifically, this paper proposes a Kinect 3D camera scheme driven by two time-series deep-learning algorithmic models: the classification and regression-based prediction models. The classification model is used to determine the respiratory phases that correspond to the lung tumor location, while the regression-based prediction model is used to predict the lung tumor displacement. Both the classification model and the prediction model were validated by testing with four categories of datasets: patient-based datasets with regular (category I) and irregular (category II) breathing patterns; and pseudopatient-based datasets with regular (category III) and irregular (category IV) breathing patterns. In this study, the respiratory-phase classification performance of the time-series deep-learning classification model was determined by the total accuracy (the average F1 score), and the performance of the regression-based model for the prediction of lung tumor displacement was determined by the mean squared error, the mean absolute error, and the coefficient of determination (R^2^).

## 2. Study Data and Dataset Preparation

In this research, the datasets for training and testing the time-series deep-learning algorithmic models were based on existing data on patients with lung cancer (i.e., patient-based datasets) and on those acquired from the dynamic thorax phantom (pseudopatient-based datasets). This paper proposes two time-series deep-learning algorithmic models: a deep-learning classification model (the classification model) and a regression-based prediction model (the prediction model). The deep-learning classification model determines the respiratory phase that corresponds to the lung tumor location, while the deep-learning regression-based model predicts the lung tumor displacement. 

### 2.1. Acquisition and Preparation of the Patient-Based Datasets 

The patient-based input (feature) and output (target) datasets for training and testing the time-series deep-learning classification and regression-based prediction models were acquired from an existing database of 40 lung cancer patients. The patients were 45–65 years of age with a body weight of 50–80 kg and respiratory rates of 12–30 breaths per minute (bpm). Respiratory rates below 12 bpm, between 12 and 20 bpm, and above 20 bpm at rest a indicate low, normal, and high breathing rate, respectively [21]. Of the 40 patients with lung cancer, 20 patients exhibited a regular breathing pattern and the other 20 patients exhibited an irregular breathing pattern [22]. The use of the clinical data was reviewed and approved by the Siriraj Institutional Review Board with the Certificate of Approval (COA) no. Si 652/2021.

**Figure 1:**
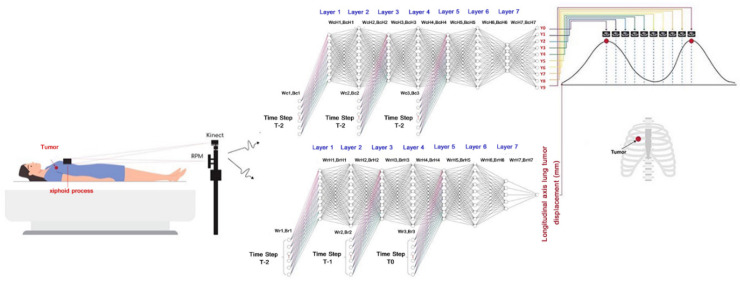
The acquisition of external chest wall movement datasets using the RPM system or the Kinect camera and a six-dot marker block.

To obtain the patient-based output (target) dataset for training and testing the proposed classification and prediction models, this research utilized 400 4D CT images of lung cancer movement in relation to the breathing pattern. The 4D CT images were generated by integrating 3D CT images of the cancer patients’ thorax with the patients’ external chest wall movement generated by the real-time position management™ (RPM) system (Varian Medical Systems, Palo Alto, CA, USA). In practice, external chest wall movement data are acquired by using the RPM system and a six-dot marker block placed on top of the xiphoid process, as shown in Figure 1. The xiphoid process is the cartilaginous section at the lower end of the sternum and is not attached to any ribs.

The 4D CT images were reconstructed using a phase-based respiratory binning method. The 3D CT images were acquired using the SOMATOM Confidence^®^ 32-slice CT simulator (Siemens, Erlangen, Germany) in the helical scanning mode and 120 kV, 240 mA, a 3 mm slice thickness, and a 0.5 s gantry rotation.

**Figure 2:**
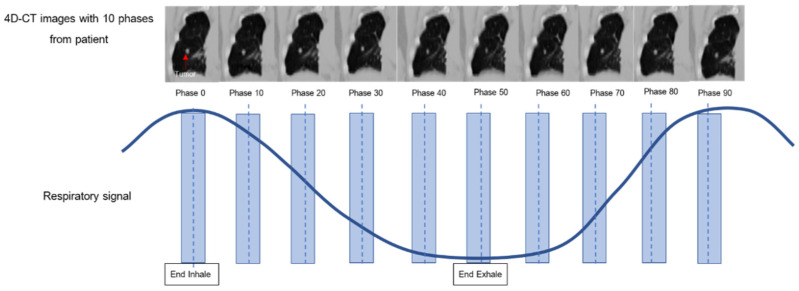
The 10 respiratory phases and the corresponding 4D CT images with the lung tumor.

Specifically, this research systematically selected 10 breathing cycles per patient [23], where a single breathing cycle consisted of an inhalation and an exhalation (i.e., one inhalation plus one exhalation is one single breathing cycle). Each breathing cycle was subsequently segregated into 10 respiratory phases, consisting of phases 0%, 10%, 20%, 30%, 40%, 50%, 60%, 70%, 80%, and 90%, where the 0%, 10–40%, 50%, and 60–90% respiratory phases represent the end-inhale, mid-exhale, end-exhale, and mid-inhale phases, respectively, as shown in Figure 2. Given the 40 lung cancer patients (20 patients each with regular and irregular breathing patterns), the total number of images in each dataset was 400 (10 respiratory phases per patient × 40 patients with lung cancer), consisting of 200 image datasets each for the patients with regular and irregular breathing patterns.

The 10 respiratory phases associated with each lung cancer patient were used as the output (target) of the time-series deep-learning classification model. Meanwhile, the output (target) of the time-series deep-learning regression-based prediction model is the lung tumor displacement (in millimeters). To obtain the output (target) of the regression-based prediction model, this research enlisted the assistance of a radiation oncologist to perform tumor localization and a medical physicist to determine the lung tumor displacement based on the 400 4D CT images. The tumor localization was manually carried out using the syngo.via imaging software (Siemens, Erlangen, USA), with a window width and level of 1500–2000 HU and 450–600 HU, respectively. The Hounsfield unit (HU) is a relative quantitative measurement of radio density used by radiologists in the interpretation of CT images.

In addition, to obtain the patient-based input (feature) dataset for training and testing the proposed classification and prediction models, this research relied on the patients’ external chest wall movement data generated by the RPM system (i.e., the 40 lung cancer patients’ chest wall movement), consisting of 400 RPM datasets. Since this research aimed to replace the costly RPM system with a highly efficient and budget-friendly Kinect v2 3D camera (Microsoft Inc., Redmond, WA, USA), a dynamic thorax phantom (Model 008A, CRIS, Norfolk, VA, USA) was utilized whereby the RPM datasets (belonging to the 40 lung cancer patients) were programmed into the dynamic phantom to mimic the external chest wall movement of the patients. The chest wall movements of the dynamic phantom were then tracked by the Kinect v2 3D camera and the six-dot external marker block (Figure 1), giving rise to 400 Kinect-generated chest wall movements.

**Figure 3:**
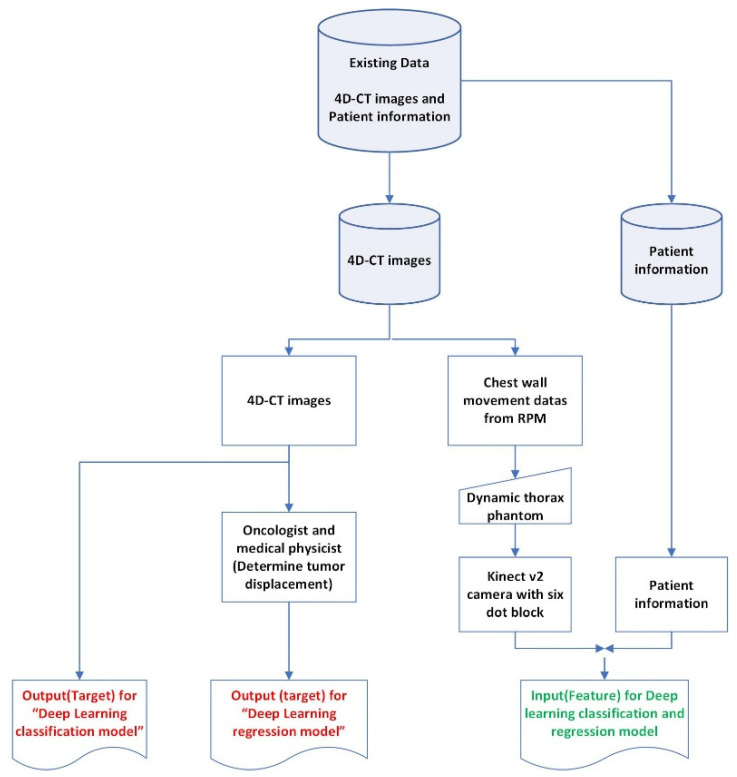
The diagram of the acquisition and preparation of the patient-based datasets.

To further enhance the performance of the time-series deep-learning classification and regression-based prediction models, the input (feature) dataset for training and testing both models also incorporated the patients’ specifics, including the lung cancer patients’ age, weight, height, breaths per minute (bpm), heart rate (HR), and midpoint of the cancer position (x, y, z) [24] in addition to the Kinect-generated chest wall movement data. In this research, the midpoint of the cancer position (x, y, z) was used as an input (feature) in place of the size of the lung tumor. Figure 3 shows the diagram of the acquisition and preparation of the patient-based datasets.

Specifically, out of the 400 patient-based input (feature) datasets, 200 datasets (100 each for patients with regular and irregular breathing patterns) were used to train the time-series deep-learning classification model and also the regression-based prediction model, while the remaining 200 patient-based input (feature) datasets were used to test the classification model and the regression-based prediction model. 

Moreover, there were two groups of 400 corresponding patient-based output (target) datasets: the 0–90% respiratory phases for the first grouping (400 datasets) and the lung tumor displacement for the second grouping (400 datasets). For the proposed classification model, the respiratory-phase output (target) datasets (200 datasets) were used to train the classification model, while the remaining 200 respiratory-phase output (target) datasets were used to test the classification model. For the regression-based prediction model, the tumor-displacement output (target) datasets (200 datasets) were used to train the prediction model, while the remaining 200 tumor-displacement output (target) datasets were used to test the regression-based prediction model.

### 2.2. Acquisition and Preparation of the Pseudopatient-Based Datasets

To enhance the classification and prediction performance of both time-series deep-learning models, this research deliberately created an additional dataset of external chest wall movements using the dynamic thorax phantom (i.e., 6000 pseudopatient-based datasets). In this research, ‘pseudopatients’ refer to the dynamic thorax phantom with a lung tumor programmed with varying breathing patterns and breaths per minute. 

To derive the additional pseudopatient-based dataset, a total of 6000 data points of external chest wall movement, varying by the breathing patterns (30 pseudopatients with regular breathing and 30 pseudopatients with irregular breathing) and breaths per minute (below 12, between 12 and 20, and above 20 bpm), were first created. The 6000 data points were then programmed into the dynamic thorax phantom to generate the corresponding 6000 pseudopatient-based datasets, consisting of 3000 datasets each for pseudopatients with regular and irregular breathing patterns. In addition, the 3000 datasets of the pseudopatients with regular breathing were divided into 1000 datasets each for below 12, between 12 and 20, and above 20 bpm. Similarly, the 3000 datasets of the pseudopatients with irregular breathing were divided into 1000 datasets each for below 12, between 12 and 20, and above 20 bpm. 

The pseudopatient-based output (target) dataset for training and testing the proposed classification and prediction models was comprised of 6000 4D CT images of the lung cancer movement in relation to the breathing pattern. The 4D CT images were rendered by integrating 3D CT images of the dynamic thorax phantom with the corresponding external chest wall movement generated by the Kinect v2 3D camera.

Given the 60 pseudopatients (30 each with regular and irregular breathing patterns), the total number of images in the pseudopatient-based output (target) dataset was 6000 (10 respiratory phases per patient × 60 pseudopatients × repeat 10 times), consisting of 3000 image datasets each for the pseudopatients with regular and irregular breathing patterns. The 10 respiratory phases associated with each pseudopatient were used as the output (target) of the time-series deep-learning classification model, consisting of phases 0%, 10%, 20%, 30%, 40%, 50%, 60%, 70%, 80%, and 90%.

The output (target) of the time-series deep-learning regression-based prediction model is the lung tumor displacement (in millimeters). To obtain the output (target) of the regression-based prediction model, this research enlisted the assistance of a radiation oncologist to perform tumor localization using the syngo.via imaging software and a medical physicist to determine the lung tumor displacement based on the 6000 4D CT images. 

**Figure 4:**
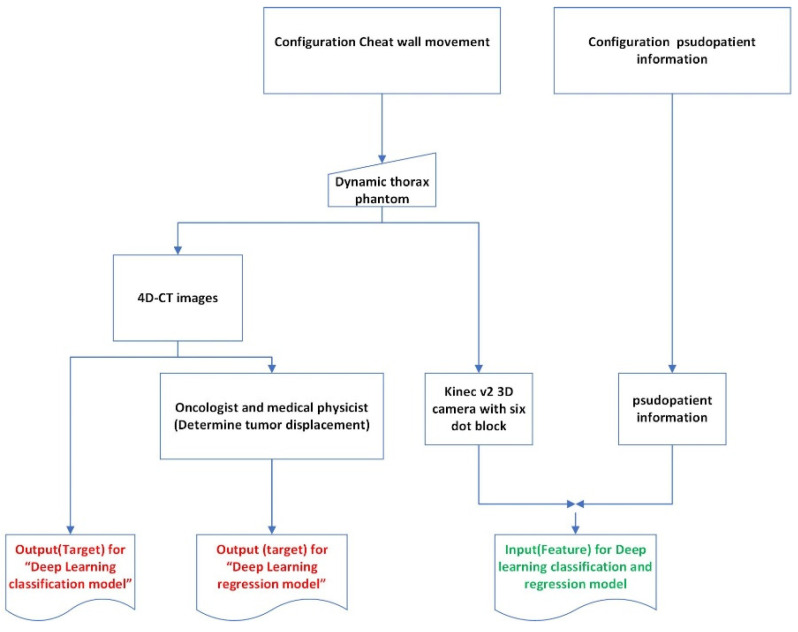
The diagram of the acquisition and preparation of the pseudopatient-based datasets.

Meanwhile, the pseudopatient-based input (feature) dataset for training and testing both models was the pseudopatients’ external chest wall movements tracked by the Kinect v2 3D camera and the six-dot marker block placed on top of the thorax phantom, giving rise to 6000 Kinect-generated chest wall movement datasets. To further enhance the performance of the time-series deep-learning classification and regression-based prediction models, the input (feature) datasets for training and testing both models also incorporated the pseudopatients’ specifics, including age, weight, height, bpm, HR, and midpoint of the cancer position (x, y, z) [24], in addition to the Kinect-generated chest wall movement data. Figure 4 shows the diagram of the acquisition and preparation of the pseudopatient-based datasets. 

Specifically, of the 6000 pseudopatient-based input (feature) datasets, 4000 datasets (2000 each for patients with regular and irregular breathing) were used to train the time-series deep-learning classification and regression-based prediction models, while the remaining 2000 pseudopatient-based input (feature) datasets were used to test the classification and regression-based prediction models. Moreover, there were two groups of 6000 corresponding pseudopatient-based output (target) datasets: the respiratory phases for the first group (6000 datasets) and the lung tumor displacement for the second group (6000 datasets). For the classification model, the respiratory-phase output (target) datasets (4000 datasets) were used to train the classification model, while the remaining 2000 respiratory-phase output (target) datasets were used to test the classification model. For the time-series deep-learning regression-based prediction model, the tumor-displacement output (target) datasets (4000 datasets) were used to train the prediction model, while the remaining 2000 tumor-displacement output (target) datasets were used to test the regression-based prediction model.

### 2.3. Training and Testing Datasets of Both Time-Series Deep-Learning Algorithmic Models

In this research, the total number of input (feature) datasets for training the time-series deep-learning classification and regression-based prediction models was identical (i.e., 4200 datasets for each model), consisting of 200 patient-based datasets (100 each for regular and irregular breathing patterns) and 4000 pseudopatient-based datasets (2000 each for regular and irregular breathing patterns). The total number of input (feature) datasets for testing the proposed classification and regression-based prediction models was also identical (i.e., 2200 datasets for each model), consisting of 200 patient-based datasets (100 each for regular and irregular breathing patterns) and 2000 pseudopatient-based datasets (1000 each for regular and irregular breathing patterns).

Moreover, the total number of output (target) datasets for training the classification and regression-based prediction models was identical (i.e., 4200 datasets for each model), consisting of the corresponding 200 patient-based output datasets and 4000 pseudopatient-based output datasets. The total number of output (target) datasets for testing the classification and regression-based prediction models was also identical (i.e., 2200 datasets for each model), consisting of 200 patient-based datasets and 2000 pseudopatient-based datasets. Nevertheless, the output (target) datasets of the classification model were the 0–90% respiratory phases, while those of the prediction model were the lung tumor displacements.

**Figure 5:**
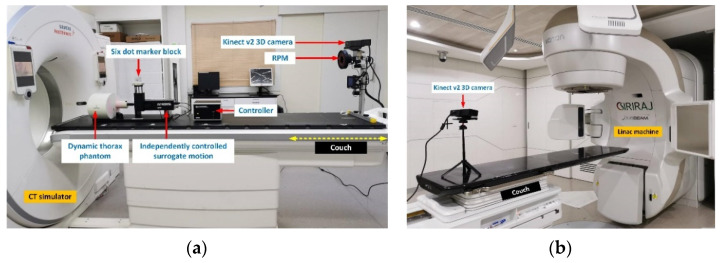
(**a**) Equipment setup used to collect datasets for training and testing the proposed time-series deep-learning models, and (**b**) the medical linear accelerator (LINAC) used to deliver high-energy X-rays for cancer treatment.

Figure 5a depicts the equipment setup used to collect the patient- and pseudopatient-based datasets for training and testing the time-series deep-learning classification and prediction models. The data collection was carried out by using the CT simulator, the dynamic thorax phantom, and the external surrogate device. Upon the completion of the training and testing, the Kinect camera scheme driven by the deep-learning classification and regression-based prediction algorithms (i.e., the time-series deep-learning Kinect scheme) would be utilized with a medical linear accelerator (LINAC) and another Kinect v2 3D camera with the six-dot marker block to individualize the delivery of high-energy X-rays or electrons in cancer treatment, as shown in Figure 5b. A medical LINAC is the device commonly used for external beam radiation treatments for patients with cancer. With the proposed time-series deep-learning-driven Kinect scheme, external beam therapy (i.e., a LINAC machine) could be designed in such a way that it destroys the cancer cells with pinpoint accuracy while sparing the nearby healthy tissue.

**Figure 6:**
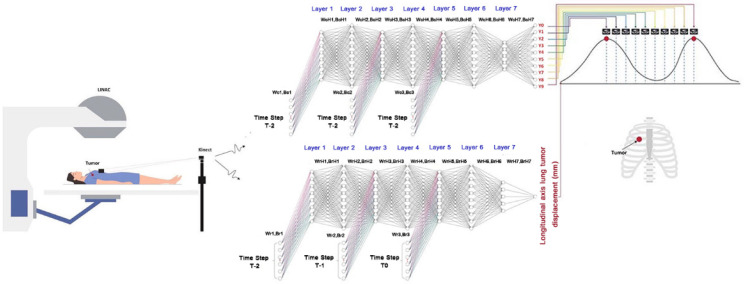
The implementation of the proposed time-series deep-learning Kinect camera scheme with the medical LINAC for treating lung cancer, where the upper and lower algorithmic models represent the classification and regression-based prediction models, respectively.

Figure 6 illustrates the implementation of the proposed time-series deep-learning Kinect v2 3D camera scheme with the medical LINAC machine for treating lung cancer, corresponding to Figure 5b. In Figure 6, the upper algorithmic scheme represents the time-series deep-learning classification model, and the lower algorithmic scheme represents the time-series deep-learning regression-based prediction model. The purpose of the classification model is to determine the respiratory phase that corresponds to the lung tumor location, and the purpose of the deep-learning regression-based model is to predict the lung tumor displacement.

## 3. Time-Series Deep-Learning Algorithmic Models

Respiratory motion is oscillatory in nature. Therefore, with a single isolated sample, there will be no distinction between inhaling and exhaling. In order to make accurate prediction, a series of samples has to be taken into account. Time series analysis can provide the consequences of and insights into the given dataset’s features that change over time-supporting the prediction of the future values of the time series variable. This paper proposes two time-series deep-learning algorithmic models: a classification model and a regression-based prediction model. The time-series deep-learning classification model is used to determine the respiratory phase that corresponds to the lung tumor location, and the time-series deep-learning regression-based model is used to predict the lung tumor displacement.

### 3.1. The Time-Series Deep-Learning Classification Model

**Figure 7:**
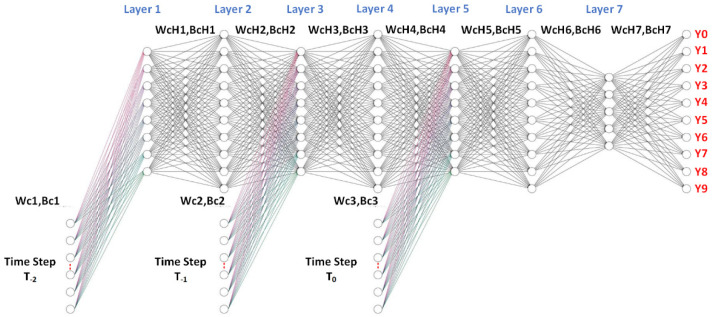
Time-series deep-learning algorithmic scheme for respiratory phase classification.

Figure 7 illustrates the time-series deep-learning algorithmic scheme for classification of the respiratory phase with a lung tumor. As described above, each breathing cycle (i.e., an inhalation and an exhalation) was segregated into 10 respiratory phases. As a result, the output (target) of the time-series deep-learning classification model consists of 10 respiratory phases (Y0–Y9).

In Figure 7, the patient- and pseudopatient-based input (feature) datasets at time T_0_ (i.e., the current period), T_−1_, and T_−2_ are fed into the respective input nodes. Each time period comprises 11 input nodes, consisting of the x, y, z coordinates from the six-dot marker block (3 features) and patients’ specifics (8 features, including age, weight, height, breaths per minute, heart rate, and the x, y, z coordinates of the midpoint of the tumor position). The T_−2_, T_−1_, and T_0_ input (feature) datasets were independently fed into hidden layers 1, 3, and 5, given as Wc_1_, Bc_1_; Wc_2_, Bc_2_; and Wc_3_, Bc_3_, respectively, where Wc and Bc are the weight and bias coefficients of the input (feature) of the classification model, respectively. In the training process, Wc_1_, Bc_1_; Wc_2_, Bc_2_; and Wc_3_, Bc_3_ were optimized by the gradient descent iterative optimization algorithm with a learning rate (α) and an epoch of 0.1 and 1000, respectively. In addition, to avoid the gradient vanishing problem, shared weights and biases were used for Wc_1_, Bc_1_; Wc_2_, Bc_2_; and Wc_3_, Bc_3_.

The algorithmic scheme for classification consists of seven hidden layers, with 8, 10, 8, 10, 8, 10, and 5 nodes in the first, second, third, fourth, fifth, sixth, and seventh hidden layers, respectively. In the training process, the weight (WcH) and bias (BcH) of the hidden layers (i.e., WcH_1_, BcH_1_; WcH_2_, BcH_2_; WcH_3_, BcH_3_; WcH_4_, BcH_4_; WcH_5_, BcH_5_; WcH_6_, BcH_6_; and WcH_7_, BcH_7_) were optimized by the gradient descent iterative optimization algorithm with an α and an epoch of 0.1 and 1000, respectively. Furthermore, L1-norm regularization was used to avoid overfitting, and the iteration procedure was terminated once the cross-entropy loss of the training and testing datasets diverged.

In the output (target) layer, there were 10 output nodes (Y0–Y9), corresponding to the 10 respiratory phases with a lung tumor (i.e., phases 0%, 10%, 20%, 30%, 40%, 50%, 60%, 70%, 80%, and 90%), with *SoftMax* as the activation function. The outputs of the time-series deep-learning classification model are given as probabilistic values. 

The rationale behind the incorporation of time series into the classification algorithmic scheme is to prevent the algorithm from returning erroneous respiratory phases. Specifically, in the absence of time series, the algorithmic scheme could misidentify the respiratory phase. For example, without time series, the respiratory phase Y1 (i.e., phase 10%) could be erroneously identified as Y9 (phase 90%), Y2 (phase 20%) as Y8 (phase 80%), and Y3 (phase 30%) as Y7 (phase 70%).

Prior to training and testing the time-series deep-learning classification algorithm, the patient- and pseudopatient-based input (feature) and output (target) training datasets and the corresponding testing datasets were normalized using standardization (Equation (1)):

(1) $$\mathrm{Standardization}=\frac{\mathrm{Dataset}-\mathrm{Mean}\;\mathrm{of}\;\mathrm{Dataset}}{\mathrm{SD}}$$

where Dataset is the input and output dataset (i.e., X_train_, Y_train_, X_test_, Y_test_), Mean of Dataset is the mean value of the input and output datasets, and *SD* is the standard deviation.

In the feedforward, the hyperbolic tangent function (tanh(z)) is the activation function between hidden layers, as expressed in Equation (2), where tanh(z) = [−1, 1]. The activation function Softmax(z) was used in the output layer, as expressed in Equation (3) [25], where z is the linear combination, as expressed in Equations (4) and (5). Equation (4) is for hidden layers 1, 2, 4, 6, 7, and 8., while Equation (5) is for hidden layers 3 and 5. The outputs of the time-series deep-learning classification model are given as probabilistic values.

(2) $$\tan h\left(z\right)=\frac{\left(e^{z}-e^{-z}\right)}{\left(e^{z}+e^{-z}\right)}$$

(3) $${\hat{Y}}_{n}=\mathrm{Softmax}\left(z\right)=\frac{e^{z_{i}}}{\sum_{j=1}^{k}e^{z_{j}\;}}$$

(4) $$Z_{H\left(1,2,4,6,7\right)}=[\begin{array}{c} Z_{1}\\ Z_{2}\\ \vdots \\ Z_{N} \end{array}]=\left[\begin{array}{ccc} \begin{array}{c} x_{1}^{1}w_{c}{}_{1}\\ x_{2}^{1}w_{c1}\\ \begin{array}{c} \vdots \\ x_{N}^{1}w_{c1} \end{array} \end{array} & \begin{array}{c} x_{1}^{2}w_{c2}\\ x_{2}^{2}w_{c2}\\ \begin{array}{c} \vdots \\ x_{N}^{2}w_{c2} \end{array} \end{array} & \begin{array}{cc} \begin{array}{c} \ldots \\ \ldots \\ \begin{array}{c} \vdots \\ \ldots \end{array} \end{array} & \begin{array}{c} x_{1}^{D}w_{\mathrm{cD}}\\ x_{2}^{D}w_{\mathrm{cD}}\\ \begin{array}{c} \vdots \\ x_{N}^{D}w_{\mathrm{cD}} \end{array} \end{array} \end{array} \end{array}\right]+[\begin{array}{cccc} B_{c1} & B_{c2} & \ldots & B_{cD} \end{array}]$$

(5) $$Z_{H\left(3,5\right)}={\left[\begin{array}{ccc} \begin{array}{c} x_{1}^{1}w_{c}{}_{1}\\ x_{2}^{1}w_{c1}\\ \begin{array}{c} \vdots \\ x_{N}^{1}w_{c1} \end{array} \end{array} & \begin{array}{c} x_{1}^{2}w_{c2}\\ x_{2}^{2}w_{c2}\\ \begin{array}{c} \vdots \\ x_{N}^{2}w_{c2} \end{array} \end{array} & \begin{array}{cc} \begin{array}{c} \ldots \\ \ldots \\ \begin{array}{c} \vdots \\ \ldots \end{array} \end{array} & \begin{array}{c} x_{1}^{D}w_{\mathrm{cD}}\\ x_{2}^{D}w_{\mathrm{cD}}\\ \begin{array}{c} \vdots \\ x_{N}^{D}w_{\mathrm{cD}} \end{array} \end{array} \end{array} \end{array}\right]}_{\left\{x={\;x}_{0,-1}\right\}\;}+Z_{H\left(2,4\right)}$$

In the backpropagation, the cross-entropy between the normalized training output dataset (Y_train_; Y_n_) and the predicted normalized output (${\hat{Y}}_{n}$) is first calculated using Equation (6) [26]:

(6) $$J\left(w\right)=-\frac{1}{N}\sum_{n=1}^{N}(Y_{n}\log({\hat{Y}}_{n}))$$

where $Y_{n}$ is the actual output probabilistic value and ${\hat{Y}}_{n}$ is the predicted output probabilistic value. The gradient descent iterative optimization algorithm was applied to optimize W and B (Wc_1_, Bc_1_; Wc_2_, Bc_2_; Wc_3_, Bc_3_; WcH_1_, BcH_1_; WcH_2_, BcH_2_; WcH_3_, BcH_3_; WcH_4_, BcH_4_; WcH_5_, BcH_5_; WcH_6_, BcH_6_; and WcH_7_, BcH_7_) by using Equation (7) [26] and the chain rule derivative.

(7) $$\frac{\partial J\left(w\right)}{\partial W_{i}}\;\mathrm{and}\;\frac{\partial J\left(w\right)}{\partial B_{i}}$$

where i = 1, 2, 3, 4, 5, 6, and 7 corresponding to Wc_1_, Bc_1_; Wc_2_, Bc_2_; Wc_3_, Bc_3_; WcH_1_, BcH_1_; WcH_2_, BcH_2_; WcH_3_, BcH_3_; WcH_4_, BcH_4_; WcH_5_, BcH_5_; WcH_6_, BcH_6_; and WcH_7_, BcH_7_. The derivative of the tanh(z) activation function for hidden layers is expressed in Equation (8).

(8) $$\frac{\partial \left[\tan h\left(z\right)\right]}{\partial z}=1-{\left(\tanh(z)\right)}^{2}$$

The performance of the time-series deep-learning classification model was assessed by the F1 score and the total accuracy (the average of the F1 scores) [26]. The F1 score is a value that indicates the classification performance of an algorithmic model based on Precision (Equation (9)) and Recall (Equation (10)). The F1 score and the total accuracy (the average of the F1 scores) can be calculated by Equations (11) and (12), respectively.

(9) $$\mathrm{Precision}=\frac{\mathrm{TP}}{\mathrm{TP}+\mathrm{FP}}$$

(10) $$\mathrm{Recall}\;=\frac{\mathrm{TP}}{\mathrm{TP}+\mathrm{FN}}$$

where TP is the number of true positives, FP is the number of false positives, and FN is the number of false negatives. 

In this research, a true positive (TP) means that the time-series deep-learning classification model is able to correctly determine the respiratory phase with a lung tumor. A false positive (FP) means that the time-series deep-learning classification model erroneously determines the respiratory phase with a lung tumor. For example, the actual respiratory phase with a lung tumor is phase 10% but the classification model returns any other respiratory phase (i.e., phases 0%, 20%, 30%, 40%, 50%, 60%, 70%, 80%, and 90%) than phase 10%. A false negative (FN) means that the time-series deep-learning classification model erroneously determines the respiratory phase. For example, in an FN (which is contrary to an FP), the classification model returns the respiratory phase 10% although the actual respiratory phase with the lung tumor is another phase (phase 0%, 20%, 30%, 40%, 50%, 60%, 70%, 80%, or 90%).

(11) $$F1\;\mathrm{Score}=2\times \frac{\mathrm{Precision}*\mathrm{Recall}}{\mathrm{Precision}+\mathrm{Recall}}$$

(12) $$\mathrm{Total}\;\mathrm{Accuracy}\;=\frac{\left(F1\;\mathrm{Score}\;\left(Y0\right)\right)+\ldots +\left(F1\;\mathrm{Score}\;\left(Y\;9\right)\right)}{10}$$

### 3.2. The Time-Series Deep-Learning Regression-Based Prediction Model

**Figure 8:**
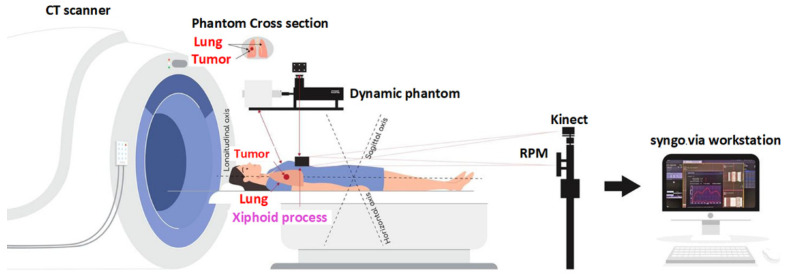
The acquisition of respiratory phases and external chest wall movements along the human anatomical axes (the longitudinal, horizontal, and sagittal axes).

Figure 8 shows the time-series deep-learning regression-based algorithmic scheme for prediction of the lung tumor displacement. Specifically, the proposed time-series deep-learning regression-based prediction model is used to predict the lung tumor displacement along the longitudinal axis (in the head–toe direction). The proposed time-series deep-learning prediction model is applicable to predicting the lung tumor displacement in the head–toe direction because the lung tumor motility is predominantly along the longitudinal axis [10].

**Figure 9:**
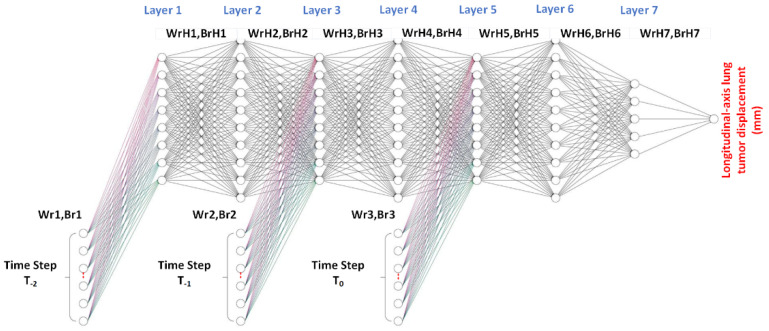
Time-series deep-learning regression-based algorithmic scheme for prediction of lung tumor displacement.

In Figure 9, the patient- and pseudopatient-based input (feature) datasets at time T_0_, T_−1_, and T_−2_ are fed into the respective input nodes. Each time period comprises 11 input nodes, consisting of the x, y, z coordinates from the six-dot marker block (3 features) and patients’ specifics (8 features, including age, weight, height, breaths per minute, heart rate, and the x,y,z coordinates of the midpoint of lung tumor). The T_−2_, T_−1_, and T_0_ input (feature) datasets were independently fed into hidden layers 1, 3, and 5, given as Wr_1_, Br_1_; Wr_2_, Br_2_; and Wr_3_, Br_3_, respectively, where Wr and Br are the weight and bias coefficients of the input (feature) of the regression-based prediction model. In the training process, Wr_1_, Br_1_; Wr_2_, Br_2_; and Wr_3_, Br_3_ were optimized by the gradient descent iterative optimization algorithm with a learning rate (α) and an epoch of 0.1 and 5000, respectively. In addition, to avoid the gradient vanishing problem, shared weights and biases were used for Wr_1_, Br_1_; Wr_2_, Br_2_; and Wr_3_, Br_3_.

The regression-based algorithmic scheme comprises seven hidden layers, with 8, 10, 8, 10, 8, 10, and 5 nodes in the first, second, third, fourth, fifth, sixth, and seventh hidden layers, respectively. In the training process, the weight (WrH) and bias (BrH) of the hidden layers (i.e., WrH_1_, BrH_1_; WrH_2_, BrH_2_; WrH_3_, BrH_3_; WrH_4_, BrH_4_; WrH_5_, BrH_5_; WrH_6_, BrH_6_; and WrH_7_, BrH_7_) were optimized by the gradient descent iterative optimization algorithm with an α and an epoch of 0.1 and 5000, respectively. Furthermore, L1-norm regularization was used to avoid overfitting and the iteration procedure was terminated once the cross-entropy loss of the training and testing datasets diverged.

The output (target) layer is the longitudinal-axis lung tumor displacement, with the rectified linear unit (ReLU) as the activation function. The output of the time-series deep-learning regression-based prediction model is the longitudinal-axis lung tumor displacement (in millimeters).

The rationale behind the incorporation of time series into the algorithmic scheme is to prevent the algorithm from returning an erroneous tumor displacement. In the absence of time series, the algorithmic scheme could miscalculate the lung tumor displacement relative to the reference point (i.e., phase 50% or the trough of the respiration).

Prior to training and testing the time-series deep-learning regression-based prediction model, the training and testing input and output datasets ($X_{\mathrm{train}}$, $Y_{\mathrm{train}}$, $X_{\mathrm{test}}$, $Y_{\mathrm{test}}$) were normalized using min–max normalization (Equation (13)).

(13) $${\mathrm{Data}}_{\mathrm{normalization}}=\frac{\left(\mathrm{Dataset}-{\mathrm{Dataset}}_{\min}\right)}{\left({\mathrm{Dataset}}_{\max}-{\mathrm{Dataset}}_{\min}\right)}$$

where Dataset is the training and testing input and output dataset (i.e., $(X_{\mathrm{train}},Y_{\mathrm{train}},X_{\mathrm{test}},Y_{\mathrm{test}}$); Dataset_min_ is the minimum training input and output dataset $(X_{\mathrm{train}},Y_{\mathrm{train}}$) and the minimum testing input and output dataset ($X_{\mathrm{test}},Y_{\mathrm{test}}$); and Dataset_max_ is the maximum training input and output dataset $(X_{\mathrm{train}},Y_{\mathrm{train}}$) and the maximum testing input and output dataset ($X_{\mathrm{test}},Y_{\mathrm{test}}$). The value of the normalized datasets (${\mathrm{Data}}_{\mathrm{normalization}}$) lies between 0 and 1 ([0, 1]).

In the feedforward of the time-series deep-learning regression-based prediction model, the activation function between hidden layers is the hyperbolic tangent function (tanh(z)) in Equation (3), where tanh(z) = [−1, 1]. The activation function ReLU(z) was used in the output layer, as expressed in Equation (14), where z is the linear combination, as expressed in Equations (4) and (5). Equation (4) is for hidden layers 1, 2, 4, 6, 7, and 8, while Equation (5) is for hidden layers 3 and 5. 

The predicted output (${\hat{y}}_{n}$) of the time-series deep-learning regression-based prediction model is the longitudinal-axis lung tumor displacement (based on the chest wall movement tracked by the Kinect v2 3D camera and patients’ specifics, including age, weight, height, breaths per minute, HR, and midpoint of the cancer).

(14) $$\mathrm{ReLU}\left(z\right)=\{\begin{array}{c} 0,\;\;z<0\\ z,\;\;z\geq 0 \end{array}$$

In the backpropagation of the time-series deep-learning regression-based prediction model, the mean squared error (MSE) between the normalized training output dataset ($Y_{\mathrm{train}};y_{n}$) and the predicted normalized output (${\hat{y}}_{n}$) is first calculated by using Equation (15), and the gradient descent iterative optimization algorithm is subsequently applied to fine-tune W and B by using Equation (16) and the chain rule derivative.

(15) $$\mathrm{MSE}=\frac{1}{n}\;\overset{n}{\underset{i\;=1}{\sum }}{\left(y_{n}-{\hat{y}}_{n}\right)}^{2}$$

(16) $$\frac{\partial \mathrm{MSE}}{\partial W_{i}}\;\mathrm{and}\;\frac{\partial \mathrm{MSE}}{\partial B_{i}}$$

where i = 1, 2, 3, 4, 5, 6, and 7 corresponding to Wr_1_, Br_1_; Wr_2_, Br_2_; Wr_3_, Br_3_; WrH_1_, BrH_1_; WrH_2_, BrH_2_; WrH_3_, BrH_3_; WrH_4_, BrH_4_; WrH_5_, BrH_5_; WrH_6_, BrH_6_; and WrH_7_, BrH_7_. The derivative of the tanh(z) activation function for hidden layers is expressed in Equation (8).

The prediction performance of the time-series deep-learning regression-based prediction model was assessed by the mean squared error (MSE; Equation (15)), the mean absolute error (MAE; Equation (17)), and the coefficient of determination (R^2^; Equation (18)).

(17) $$\mathrm{MAE}=\frac{1}{n}\;\overset{n}{\underset{i\;=1}{\sum }}\left|y_{n}-{\hat{y}}_{n}\right|$$

where $y_{n}$ is the normalized testing output dataset ($Y_{\mathrm{test}}$), ${\hat{y}}_{n}$ is the predicted normalized output ($Y_{\mathrm{predict}}$), and n is the number of datasets.

(18) $$R^{2}=\frac{\left(\mathrm{Var}\left(Y\right)-\mathrm{MSE}\right)}{\mathrm{Var}\left(Y\right)}$$

where Var is the mean of the differences between $y_{n}$ and $\mathrm{average}(y_{n})$ squared and MSE is the mean squared error (Equation (15)). 

## 4. Results and Discussion

This section discusses the performance of the time-series deep-learning classification and prediction models under the four dataset categories (patient-based datasets with regular (category I) and irregular (category II) breathing patterns and pseudopatient-based datasets with regular (category III) and irregular (category IV) breathing patterns).

### 4.1. Classification and Prediction Performance for Patient-Based Datasets with a Regular Breathing Pattern (Category I)

Table 1 tabulates the results of the time-series deep-learning classification model of the respiratory phases for the patient-based datasets with a regular breathing pattern in terms of F1 scores and total accuracy (the average of the F1 scores). The proposed classification model correctly determined all respiratory phases with a lung tumor, as evidenced by the F1 scores of 100% for all classifications (Y0–Y9) and the total accuracy of 100%.

**Figure 10:**
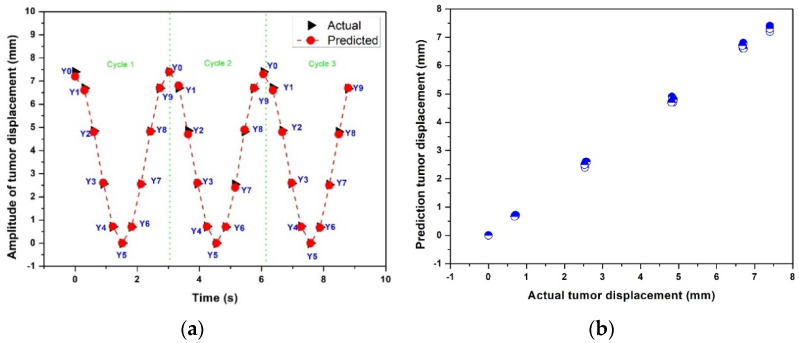
The actual and predicted results of the time-series deep-learning classification and prediction models for the patient-based datasets with a regular breathing pattern: (**a**) the respiratory phase and lung tumor displacement; (**b**) a scatter plot of the regression-based prediction model.

Figure 10a,b show the actual and predicted results of the time-series deep-learning classification and prediction models for the patient-based datasets with a regular breathing pattern. Figure 10a compares the actual and predicted respiratory phases and lung tumor displacements. The classification model correctly determined all respiratory phases with a lung tumor for three example breathing cycles, consistent with the F1 scores and total accuracy shown in Table 1. Given the space limitations, only three breathing cycles are illustrated in the figure. Moreover, the MSE, MAE, and R^2^ of the time-series deep-learning regression-based prediction model are 1.3%, 0.65%, and 0.98, respectively, indicating that the proposed prediction model can predict the lung tumor displacement with high accuracy.

Figure 10b shows the scatter plot between the actual and predicted lung tumor displacement using the time-series deep-learning regression-based prediction model. The relationship between the actual and predicted lung tumor displacement is nearly linear, suggesting that the proposed predication model can predict the lung tumor displacement with high accuracy.

### 4.2. Classification and Prediction Performance for Patient-Based Datasets with an Irregular Breathing Pattern (Category II)

Table 2 tabulates the F1 scores and total accuracy of the time-series deep-learning classification model for the patient-based datasets with an irregular breathing pattern. The proposed classification model correctly identified all respiratory phases with a lung tumor, as evidenced by the F1 scores of 100% for all classifications (Y0–Y9) and the total accuracy of 100%.

**Figure 11:**
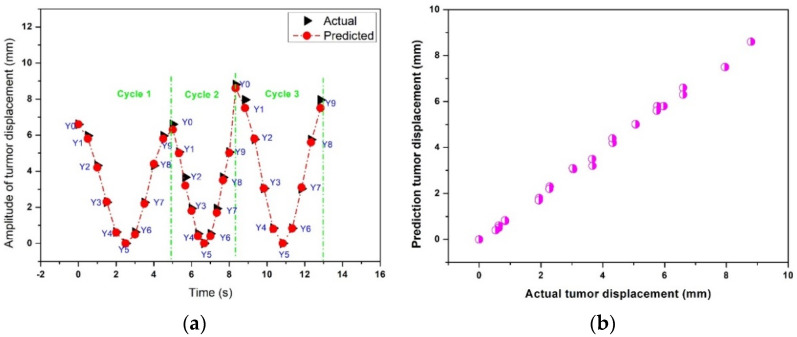
The actual and predicted results of the time-series deep-learning classification and prediction models for the patient-based datasets with an irregular breathing pattern: (**a**) the respiratory phase and lung tumor displacement; (**b**) a scatter plot of the regression-based prediction model.

Figure 11a,b show the actual and predicted results of the time-series deep-learning classification and prediction models for the patient-based datasets with an irregular breathing pattern. Figure 11a compares the actual and predicted respiratory phases and lung tumor displacements. The classification model correctly determined all respiratory phases with a lung tumor in all breathing cycles. The MSE, MAE, and R^2^ of the time-series deep-learning regression-based prediction model are 1.3%, 0.65%, and 0.98, indicating that the proposed regression-based prediction model can predict the lung tumor displacement with very high accuracy.

Figure 11b shows the scatter plot between the actual and predicted lung tumor displacement using the time-series deep-learning regression-based prediction model. The relationship between the actual and predicted lung tumor displacement is nearly linear, suggesting that the proposed prediction model can predict the lung tumor displacement with high accuracy.

### 4.3. Classification and Prediction Performance for Pseudopatient-Based Datasets with a Regular Breathing Pattern (Category III)

Table 3 presents the F1 scores and total accuracy of the time-series deep-learning classification model for the pseudopatient-based datasets with a regular breathing pattern. In this paper, ‘pseudopatients’ refer to the dynamic thorax phantom with a lung tumor programmed with varying breathing patterns and breaths per minute. The proposed classification model correctly identified all respiratory phases with a lung tumor, as evidenced by the F1 scores of 100% for all classifications (Y0–Y9) and the total accuracy of 100%.

**Figure 12:**
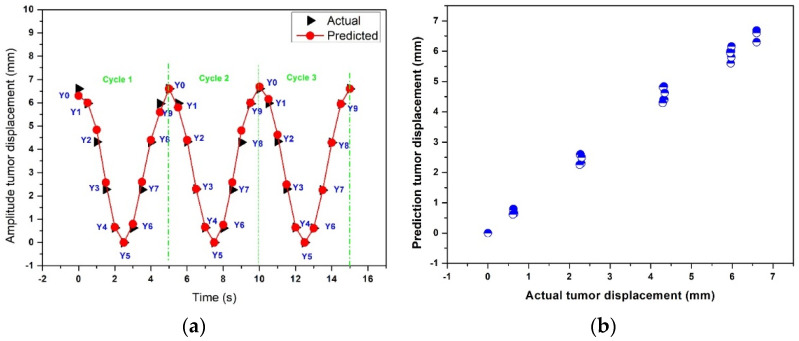
The actual and predicted results of the time-series deep-learning classification and prediction models for the pseudopatient-based datasets with a regular breathing pattern: (**a**) the respiratory phase and lung tumor displacement; (**b**) a scatter plot of the regression-based prediction model.

Figure 12a,b show the actual and predicted results of the time-series deep-learning classification and prediction models for the pseudopatient-based datasets with a regular breathing pattern. Figure 12a compares the actual and predicted respiratory phases and lung tumor displacements. The classification model correctly determined all respiratory phases with a lung tumor in all breathing cycles. The MSE, MAE, and R^2^ of the time-series deep-learning regression-based prediction model are 1.2%, 0.7%, and 0.97, indicating that the proposed regression-based prediction model can predict the lung tumor displacement with high accuracy. 

Figure 12b shows the scatter plot between the actual and predicted lung tumor displacement using the time-series deep-learning regression-based prediction model. The relationship between the actual and predicted lung tumor displacement is nearly linear, suggesting that the proposed prediction model can predict the lung tumor displacement with high accuracy.

### 4.4. Classification and Prediction Performance for Pseudopatient-Based Datasets with an Irregular Breathing Pattern (Category IV)

Table 4 shows the F1 scores and total accuracy of the time-series deep-learning classification model for the pseudopatient-based datasets with an irregular breathing pattern. The proposed classification model was able to identify most of the respiratory phases with a lung tumor correctly, except for Y3, Y4, Y6, and Y7 with respective F1 scores of 81.81%. 

**Figure 13:**
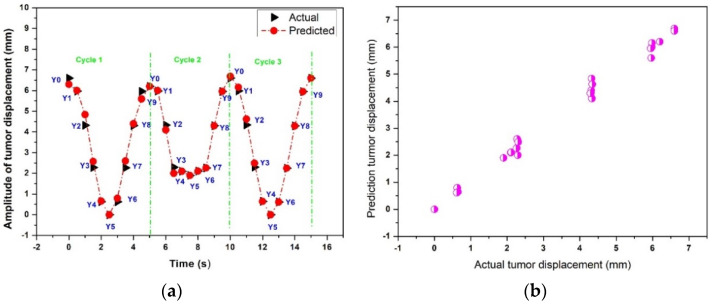
The actual and predicted results of the time-series deep-learning classification and prediction models for the pseudopatient-based datasets with an irregular breathing pattern: (**a**) the respiratory phase and lung tumor displacement; (**b**) a scatter plot of the regression-based prediction model.

The erroneous classification could be attributed to shallow breathing (as is evident in breathing cycle 2 in Figure 13), resulting in the aggregation of respiratory phases Y3 and Y4 and Y6 and Y7. The erroneous respiratory phase classification thus resulted in a total accuracy (average of the F1 scores) of 92.44%.

To circumvent the respiratory phase aggregation, the radiation oncologist is required to instruct the lung cancer patient to breathe deeply. Nevertheless, the breathing pattern (i.e., the regular and irregular breathing patterns) had no effect on the performance of the time-series deep-learning classification model, as is evidenced by the total accuracy of 100% under dataset categories I–III (Table 1, Table 2 and Table 3).

Figure 13a,b show the actual and predicted results of the time-series deep-learning classification and prediction models for the pseudopatient-based datasets with an irregular breathing pattern. Figure 13a compares the actual and predicted respiratory phases and lung tumor displacements. The classification model identified most of the respiratory phases with a lung tumor correctly, except for Y3, Y4, Y6, and Y7 with respective F1 scores of 81.81%. However, the MSE, MAE, and R^2^ of the time-series deep-learning regression-based prediction model are 1.6%, 0.8%, and 0.97, respectively, indicating that the proposed regression-based prediction model can predict the lung tumor displacement with high accuracy. 

Figure 13b shows the scatter plot between the actual and predicted lung tumor displacement using the time-series deep-learning regression-based prediction model. The relationship between the actual and predicted lung tumor displacement is less robust in comparison with those under dataset categories I, II, and III.

Regarding other studies on the prediction of tumor motion, Akimoto et al. recommend updating the 4D model several times during a treatment session to increase the accuracy of the linear regression prediction model [27]. Ginn John S et al. demonstrated that an image regression model built from a single-plane cine MRI image could be used to predict the tumor target motion for radiotherapy [28]. Zhou Dejun et al. observed that the regression-based prediction model does not represent the tumor motion accurately. CNN-driven prediction models were found to outperform the regression-based prediction model [29]. This paper proposes a Kinect v2 3D camera scheme driven by time-series deep-learning algorithmic models that can improve the accuracy of real-time tumor motion prediction compared with the regression model. Because Respiratory motion is oscillatory in nature. Therefore, with a single isolated sample, there will be no distinction between inhaling and exhaling. In order to make accurate prediction, a series of samples has to be taken into account. Time series analysis can provide the consequences of and insights into the given dataset’s features that change over time-supporting the prediction of the future values of the time series variable.

A limitation of this study is that the prediction performance may decrease with irregular breathing patterns. Additionally, predictions may be inaccurate when the motion lies outside the range of motion included in the training dataset and can be improved by increasing the number of respiratory patterns and tumor displacement measurements of prospective patients to cover any situation and input features or train the patient to breathe regularly so that the model can provide accurate predictions.

Although Kinect was developed for gaming, its performance is suitable for a range of medical applications [30]. The Kinect V2 camera can provide information on the patient’s position and the patient’s movement by tracking the body surface motion during radiotherapy. In addition, the Kinect V2 camera may be useful in other medical applications. For example, Heß et al. used the Kinect camera to determine the correlation between body surface motion and internal organs for the purpose of respiratory motion correction to reduce the blurring effect and attenuation correction artifacts in positron emission tomography (PET) images [31]. Noonan et al. modified the Kinect V3 camera for the purpose of tracking the motion of a subject in a routine clinical PET/CT scan [32]. The Kinect V3 camera is also currently the subject of a clinical trial.

## 5. Conclusions

This paper proposed an economical and highly efficient Kinect v2 3D camera scheme driven by two time-series deep-learning algorithmic models: a classification model and a regression-based prediction model. The classification model is used to determine the respiratory phases that correspond to the lung tumor location, and the regression-based prediction model is used to predict the lung tumor displacement (in millimeters). The budget-friendly Kinect v2 3D camera is employed in place of the costly RPM system. In the study, both the classification model and the prediction model were validated by testing with four dataset categories (patient-based datasets with regular (category I) and irregular (category II) breathing patterns and pseudopatient-based datasets with regular (category III) and irregular (category IV) breathing patterns). ‘Pseudopatients’ refer to the dynamic thorax phantom with a lung tumor programmed with varying breathing patterns and breaths per minute. The respiratory phase classification performance of the classification model was determined by the total accuracy (average of the F1 scores), and the performance of the regression-based model for the prediction of lung tumor displacement was determined by the MSE, MAE, and R^2^. The total accuracy was 100%, 100%, 100%, and 92.44% for the dataset categories I, II, III, and IV, respectively, with a corresponding MSE, MAE, and R^2^ of 1.2–1.6%, 0.65–0.8%, and 0.97–0.98, respectively. The numerical results indicate that both the time-series deep-learning classification and regression-based prediction models are capable of classifying the respiratory phases and predicting the lung tumor displacement with high accuracy. In comparison with the costly RPM-based scheme, the proposed time-series deep-learning Kinect 3D camera scheme is highly affordable. In addition, the time-series deep-learning classification and regression-based prediction models are both of an open-system algorithmic scheme as opposed to the closed-system technology of the RPM system. Furthermore, the proposed time-series deep-learning models were demonstrated to improve the prediction of lung tumor displacement. 

## Figures and Tables

**Table 1:** The classification performance of the time-series deep-learning classification model for patient-based datasets with a regular breathing pattern.

Classification (Phase)		Precision (Equation (9))	Recall (Equation (10))	F1 Score (Equation (11))
Y0 (phase 0%)	TP = 10	100%	100%	100%
	FP = 0			
	FN = 0			
Y1 (phase 10%)	TP = 10	100%	100%	100%
	FP = 0			
	FN = 0			
Y2 (phase 20%)	TP = 10	100%	100%	100%
	FP = 0			
	FN = 0			
Y3 (phase 30%)	TP = 10	100%	100%	100%
	FP = 0			
	FN = 0			
Y4 (phase 40%)	TP = 10	100%	100%	100%
	FP = 0			
	FN = 0			
Y5 (phase 50%)	TP = 10	100%	100%	100%
	FP = 0			
	FN = 0			
Y6 (phase 60%)	TP = 10	100%	100%	100%
	FP = 0			
	FN = 0			
Y7 (phase 70%)	TP = 10	100%	100%	100%
	FP = 0			
	FN = 0			
Y8 (phase 80%)	TP = 10	100%	100%	100%
	FP = 0			
	FN = 0			
Y9 (phase 90%)	TP = 10	100%	100%	100%
	FP = 0			
	FN = 0			
Total accuracy (average of the F1 scores; Equation (12))		100%		

Note: The definitions of TP, FP, and FN are provided in the subsection on the time-series deep-learning classification model.

**Table 2:** The classification performance of the time-series deep-learning classification model for patient-based datasets with an irregular breathing pattern.

Classification (Phase)		Precision (Equation (9))	Recall (Equation (10))	F1 Score (Equation (11))
Y0 (phase 0%)	TP = 10	100%	100%	100%
	FP = 0			
	FN = 0			
Y1 (phase 10%)	TP = 10	100%	100%	100%
	FP = 0			
	FN = 0			
Y2 (phase 20%)	TP = 10	100%	100%	100%
	FP = 0			
	FN = 0			
Y3 (phase 30%)	TP = 10	100%	100%	100%
	FP = 0			
	FN = 0			
Y4 (phase 40%)	TP = 10	100%	100%	100%
	FP = 0			
	FN = 0			
Y5 (phase 50%)	TP = 10	100%	100%	100%
	FP = 0			
	FN = 0			
Y6 (phase 60%)	TP = 10	100%	100%	100%
	FP = 0			
	FN = 0			
Y7 (phase 70%)	TP = 10	100%	100%	100%
	FP = 0			
	FN = 0			
Y8 (phase 80%)	TP = 10	100%	100%	100%
	FP = 0			
	FN = 0			
Y9 (phase 90%)	TP = 10	100%	100%	100%
	FP = 0			
	FN = 0			
Total accuracy (average of the F1 scores; Equation (12))		100%		

Note: The definitions of TP, FP, and FN are provided in the subsection on the time-series deep-learning classification model.

**Table 3:** The classification performance of the time-series deep-learning classification model for pseudopatient-based datasets with a regular breathing pattern.

Classification (Phase)		Precision (Equation (9))	Recall (Equation (10))	F1 Score (Equation (11))
Y0 (phase 0%)	TP = 100	100%	100%	100%
	FP = 0			
	FN = 0			
Y1 (phase 10%)	TP = 100	100%	100%	100%
	FP = 0			
	FN = 0			
Y2 (phase 20%)	TP = 100	100%	100%	100%
	FP = 0			
	FN = 0			
Y3 (phase 30%)	TP = 100	100%	100%	100%
	FP = 0			
	FN = 0			
Y4 (phase 40%)	TP = 100	100%	100%	100%
	FP = 0			
	FN = 0			
Y5 (phase 50%)	TP = 100	100%	100%	100%
	FP = 0			
	FN = 0			
Y6 (phase 60%)	TP = 100	100%	100%	100%
	FP = 0			
	FN = 0			
Y7 (phase 70%)	TP = 100	100%	100%	100%
	FP = 0			
	FN = 0			
Y8 (phase 80%)	TP = 100	100%	100%	100%
	FP = 0			
	FN = 0			
Y9 (phase 90%)	TP = 100	100%	100%	100%
	FP = 0			
	FN = 0			
Total accuracy (average of the F1 scores; Equation (12))		100%		

Note: The definitions of TP, FP, and FN are provided in the subsection on the time-series deep-learning classification model.

**Table 4:** The classification performance of the time-series deep-learning classification model for pseudopatient-based datasets with an irregular breathing pattern.

Classification (Phase)		Precision (Equation (9))	Recall (Equation (10))	F1 Score (Equation (11))
Y0 (phase 0%)	TP = 100	100%	100%	100%
	FP = 0			
	FN = 0			
Y1 (phase 10%)	TP = 100	100%	100%	100%
	FP = 0			
	FN = 0			
Y2 (phase2 0%)	TP = 100	100%	100%	100%
	FP = 0			
	FN = 0			
Y3 (phase 30%)	TP = 90	81.1%	81.1%	81.1%
	FP = 10 (i.e., the actual respiratory phase with a lung tumor at Y3 is incorrectly assigned to Y4)			
	FN = 10 (the actual respiratory phase with a lung tumor at Y4 is incorrectly assigned to Y3)			
Y4 (phase 40%)	TP = 90	81.1%	81.1%	81.1%
	FP = 10 (i.e., the actual respiratory phase with a lung tumor at Y4 is incorrectly assigned to Y3)			
	FN = 10 (the actual respiratory phase with a lung tumor at Y3 is incorrectly assigned to Y4)			
Y5 (phase 50%)	TP = 100	100%	100%	100%
	FP = 0			
	FN = 0			
Y6 (phase 60%)	TP = 90	81.1%	81.1%	81.1%
	FP = 10 (i.e., the actual respiratory phase with a lung tumor at Y6 is incorrectly assigned to Y7)			
	FN = 10 (the actual respiratory phase with a lung tumor at Y7 is incorrectly assigned to Y6)			
Y7 (phase 70%)	TP = 90	81.1%	81.1%	81.1%
	FP = 10 (i.e., the actual respiratory phase with a lung tumor at Y7 is incorrectly assigned to Y6)			
	FN = 10 (the actual respiratory phase with a lung tumor at Y6 is incorrectly assigned to Y7)			
Y8 (phase 80%)	TP = 100	100%	100%	100%
	FP = 0			
	FN = 0			
Y9 (phase 90%)	TP = 100	100%	100%	100%
	FP = 0			
	FN = 0			
Total accuracy (average of the F1 scores; Equation (12))		92.44%		

Note: The definitions of TP, FP, and FN are provided in the subsection on the time-series deep-learning classification model.

## Data Availability

Not applicable.
